# Impact of hypothermia on implementation of CPAP for neonatal respiratory distress syndrome in a low-resource setting

**DOI:** 10.1371/journal.pone.0194144

**Published:** 2018-03-15

**Authors:** Jennifer Carns, Kondwani Kawaza, MK Quinn, Yinsen Miao, Rudy Guerra, Elizabeth Molyneux, Maria Oden, Rebecca Richards-Kortum

**Affiliations:** 1 Department of Bioengineering, Rice University, Houston, Texas, United States of America; 2 Department of Pediatrics, College of Medicine, Queen Elizabeth Central Hospital, Blantyre, Malawi; 3 Department of Statistics, Rice University, Houston, Texas, United States of America; Hopital Robert Debre, FRANCE

## Abstract

**Background:**

Neonatal hypothermia is widely associated with increased risks of morbidity and mortality, but remains a pervasive global problem. No studies have examined the impact of hypothermia on outcomes for preterm infants treated with CPAP for respiratory distress syndrome (RDS).

**Methods:**

This retrospective analysis assessed the impact of hypothermia on outcomes of 65 neonates diagnosed with RDS and treated with either nasal oxygen (N = 17) or CPAP (N = 48) in a low-resource setting. A classification tree approach was used to develop a model predicting survival for subjects diagnosed with RDS.

**Findings:**

Survival to discharge was accurately predicted based on three variables: mean temperature, treatment modality, and mean respiratory rate. None of the 23 neonates with a mean temperature during treatment below 35.8°C survived to discharge, regardless of treatment modality. Among neonates with a mean temperature exceeding 35.8°C, the survival rate was 100% for the 31 neonates treated with CPAP and 36.4% for the 11 neonates treated with nasal oxygen (p<0.001). For neonates treated with CPAP, outcomes were poor if more than 50% of measured temperatures indicated hypothermia (5.6% survival). In contrast, all 30 neonates treated with CPAP and with more than 50% of temperature measurements above 35.8°C survived to discharge, regardless of initial temperature.

**Conclusion:**

The results of our study suggest that successful implementation of CPAP to treat RDS in low-resource settings will require aggressive action to prevent persistent hypothermia. However, our results show that even babies who are initially cold can do well on CPAP with proper management of hypothermia.

## Introduction

Neonatal hypothermia is a global challenge and is widely associated with increased risks of morbidity and mortality [1–9]. Defined by WHO as a body temperature below the normal range (36.5°C–37.5°C), hypothermia is divided into three categories: mild (36.0°C–36.4°C), moderate (32.0°C–35.9°C), and severe (<32.0°C). After delivery, the primary cause of heat loss for a newborn is evaporation of amniotic fluid [10], which can be exacerbated by convective and conductive cooling if a newborn is exposed to relatively cooler room temperatures or placed on a cold surface [11]. The full-term newborn cannot produce sufficient heat to prevent a fall in body temperature, especially on the first day of life [12], and can experience a rapid drop in body temperature at a rate of 0.2 to 1.0°C per minute upon exposure to room temperature after delivery [13]. Hypothermia can have serious health implications; temperature upon admission to the neonatal intensive care unit (NICU) is inversely related to mortality, with one large study showing a 28% increase in mortality per 1° C decrease in admission temperature [7]. Despite these well-recognized risks, hypothermia remains a pervasive problem, with an estimated 17 million newborns experiencing hypothermia annually in low-resource settings [9]. In some parts of the sub-Saharan Africa, incidence rates as high as 60–85% have been documented [[14–16]].

Preterm infants are at increased risk of hypothermia for many reasons, including higher body surface area to weight ratios, an under developed stratum corneum, low subcutaneous fat reserves, and unstable vasomotor responses that prevent adequate vasoconstriction [13]. Hypothermia is also associated with increased risk of severe respiratory distress [3], and in high income countries more than 50% of babies born at ≤31 weeks of gestation develop respiratory distress syndrome (RDS) [17].

Prevention of hypothermia and management of RDS are recognized by the WHO as essential components of newborn care [18], and are two of the key interventions for small and sick babies [19]. In high-resource settings, RDS is most often treated by providing Continuous Positive Airway Pressure (CPAP); however, many low-resource settings lack the tools to implement CPAP. Recently, several studies have described the use of CPAP in low-resource settings [20–24]. Although hypothermia is common in low-resource settings, no studies have examined the impact of hypothermia on outcomes for preterm infants treated with CPAP. As further described in the methods section, here we retrospectively analyze the impact of hypothermia on a prospective, non-randomized controlled study comparing nasal oxygen and a low-cost bubble continuous positive airway pressure (bCPAP) device to treat respiratory illness conducted at Queen Elizabeth Central Hospital in Blantyre, Malawi [24].

## Methods

### Ethics statement

The study protocol was approved by the University of Malawi College of Medicine Research and Ethics Committee (P.05/11/ 1079) and the Institutional Review Boards at Baylor College of Medicine (H-29059) and Rice University (11–198F) prior to study initiation. Written informed consent was obtained from parents or legal guardians of participants before they were enrolled in the study.

### Participants

As previously described, a prospective, non-randomized controlled study, conducted from January to October of 2012 at Queen Elizabeth Central Hospital was carried out to evaluate the efficacy and safety of a novel, low-cost bCPAP device to treat neonatal respiratory illness in a low-resource setting [24]. Neonates admitted with severe respiratory distress, weighing at least 1,000 grams who were breathing spontaneously, viable, and identified by the treating clinician as appropriate for bCPAP treatment were eligible to participate.

### Background

Two low-cost bCPAP devices were installed in the ward, and data were collected as previously described [24]. Neonates were treated with bCPAP (treatment group) if a bCPAP system and trained clinical staff were available; otherwise the neonate received the local standard of care, nasal oxygen (control group). In some cases, a neonate in the control group was transitioned from nasal oxygen to bCPAP treatment after entering the study when a bCPAP device became available. The bCPAP device, which became commercially available in 2015 as the Pumani bCPAP (Hadleigh Health Technologies), delivered a mixture of pressurized room air and oxygen from a concentrator (Airsep, New Life Intensity, 10 LPM) to the infant via Hudson binasal prongs attached to a stockinette hat. Nursing care for children treated with bCPAP included twice daily suctioning to clear the airways of mucus, and the administration of sterile nasal saline drops every four hours to reduce mucosal drying. Neonates in the control group received nasal oxygen delivered from an oxygen concentrator via standard nasal cannulae. In both the treatment and control groups, treatment was administered until the clinician determined therapy was no longer necessary. With the exception of respiratory support, all other patient care was identical for the bCPAP and control groups [24]. Vital signs were repeated one hour after recruitment (control group) or after commencing bCPAP (treatment group), and twice daily afterward until discharge or death. Neonates were monitored for progress and complications. Each participant was assigned a final primary diagnosis according to standard clinical criteria. Co-morbidities, including sepsis and jaundice, were also noted.

Data, including outcomes and vital signs, were collected from 87 eligible neonates enrolled in the study, of which 65 were diagnosed with respiratory distress syndrome (RDS). Of the 65 diagnosed with RDS, 17 were in the nasal oxygen control group and 48 in the bCPAP treatment group, including 9 who were initially treated with nasal oxygen but transitioned to bCPAP when one became available. Weight was recorded daily, and other vital signs were recorded twice daily throughout the duration of treatment. On average, neonates diagnosed with RDS were 1.2 days of age upon study registration.

A prior analysis found that rates of survival to discharge were significantly higher for neonates treated with bCPAP compared to nasal oxygen (p = 0.018), with greatest rates of improvement (p = 0.006) for premature babies suffering from respiratory distress syndrome (RDS) [24]. In the original analysis, logistic regression was performed to identify demographic and clinical covariates that were correlated with survival; five covariates were correlated with survival, including a primary diagnosis of RDS, a co-morbidity of sepsis, birthweight, a birthweight in the very low birthweight range, and gestational age. Although some vital signs (respiratory rate, heart rate, oxygen saturation) were included in the original analysis, neonatal temperature was not included. Here, we analyze data collected in this study to further examine the role of clinical and demographic covariates, including temperature and the presence of hypothermia, on outcomes for the 65 neonates diagnosed with RDS treated with either nasal oxygen or bCPAP. Table 1 summarizes data collected for each neonate enrolled in the original study and included in this analysis.

**Table 1:** Demographic data for neonates meeting eligibility criteria.

	Nasal Oxygen	bCPAP	
		Transitioned from nasal oxygen to bCPAP	bCPAP
**Number of subjects meeting eligibility criteria diagnosed with RDS**	** 17**	**9 **	**39 **
**Gender**			
% Male	52.9%	44.4%	56.4%
**Gestational Age**			
Average (weeks)	31.5 weeks	32.0 weeks	31.5 weeks
Unknown (%)	17.6%	0.0%	7.5%
**Average Birth Weight (kg)**	**1.31 kg**	**1.37 kg**	**1.48 kg**
Very Low Birth Weight (> = 1.0kg—<1.5 kg) (%)	76.5%	77.8%	56.4%
Low Birth Weight (> = 1.5kg—<2.5 kg) (%)	23.5%	22.2%	43.6%
**Average Weight at Study Registration (kg)**	**1.31 kg**	**1.35 kg**	**1.45 kg**
Very Low (> = 1.0kg—<1.5 kg) (%)	76.5%	77.8%	64.1%
Low (> = 1.5kg—<2.5 kg) (%)	23.5%	22.2%	35.9%
**Location of birth**			
Queen Elizabeth Central Hospital (QECH) (%)	70.6%	55.6%	53.8%
Outside QECH (%)	17.6%	44.4%	33.3%
Unknown (%)	11.8%	0.0%	12.8%
**Singletons vs. Multiples**			
Singletons (%)	41.2%	44.4%	71.8%
Multiples (%)	58.8%	44.4%	28.2%
Unknown (%)	0.0%	11.1%	0.0%
**Received bag & mask ventilation prior to therapy**			
Yes (%)	5.9%	22.2%	28.2%
Unknown (%)	11.8%	11.1%	20.5%
**HIV Status**			
Exposed (%)	17.6%	22.2%	20.5%
Unknown (%)	17.6%	22.2%	12.8%
PMTCT *			
Yes (%)	23.5%	17.6%	15.4%
**Initial vital statistics**			
Average initial temperature (°C)	35.7°C	35.7°C	35.6°C
Average initial heart rate (bpm)	142.5 bpm	147.7 bpm	137.4 bpm
Average initial respiratory rate (bpm)	55.2 bpm	54.9 bpm	54.2 bpm
Average initial oxygen saturation (%)	92.4%	91.1%	89.2%
**Mean vital statistics**			
Average mean temperature (°C)	35.9°C	36.0°C	35.9°C
Average mean heart rate (bpm)	142.5 bpm	148.0 bpm	141.0 bpm
Average mean respiratory rate (bpm)	49.1 bpm	46.1 bpm	47.7 bpm
Average mean oxygen saturation (%)	91.7%	96.0%	95.1%
Average mean weight (kg)	1.31 kg	1.38 kg	1.45 kg
Average median temperature (°C)	35.9°C	36.0°C	35.9°C
**Co-Morbidities**			
Sepsis (%)	35.3%	44.4%	41.0%

*Prevention of Mother-To-Child HIV Transmission.

### Statistical methodology and analysis

A classification tree approach was used to develop a prediction model for whether a subject diagnosed with RDS would survive to discharge. Classification trees have the advantage of being nonparametric, robust to outliers, robust to missing data and providing a hierarchical importance structure for prediction variables. The prediction variables included in this study were: treatment group, gender, gestational age, birth weight, initial weight at study registration, location of birth, singletons vs. multiples, treatment with bag and mask ventilation prior to study registration, HIV status, prevention of mother-to-child transmission (PMTCT) treatment, initial temperature, initial heart rate, initial respiratory rate, initial oxygen saturation, mean temperature, mean heart rate, mean respiratory rate, mean oxygen saturation, mean weight, and diagnosis of sepsis. The initial vital signs were taken upon study registration; the mean values were calculated as the average of the daily readings for each vital sign over the duration of treatment. The classification tree is fitted by recursively partitioning the dataset into sub-groups based on the prediction variables, whereby at each split, the algorithm selects the variable resulting in the largest difference in survival between the two sub-groups. The classification tree methodology was implemented in R [25] with the rpart package [26].

The fitted classification tree was obtained using a 10-fold cross validation with the information gain index and was not *pruned*; the rpart options were set to their default values with the exception of the minimum number of observations at each node that must exist for a subsequent split to be attempted, which was set to 10. Validation of the prediction model was performed through bagging, an averaging of multiple trees, each based on a bootstrap sample (resampling with replacement) from the original dataset. One hundred bootstrapped trees were generated, yielding 100 predictions for each of the 65 subjects. To prevent bias due to overfitting, out-of-bag predictions were used: the probability of survival for each subject was estimated from bootstrapped trees constructed without that observation.

As described in the results, regression tree analysis indicated that hypothermia played an important role in outcomes for neonates diagnosed with RDS, regardless of whether they received treatment with bCPAP or nasal oxygen. To further examine the role of hypothermia on outcomes, we compared survival to discharge for neonates in the treatment and control groups, stratified by mean temperature, by initial temperature, and by the fraction of time the neonate’s temperature was greater than or equal to 35.8° C. When indicated, differences in survival between groups were assessed using a one-sided Fisher’s exact test, and differences between continuous variables using a two sided t-test for equality of means (unequal variances assumed). Results for all analyses were considered significant at the 5% level.

## Results

**Fig 1:**
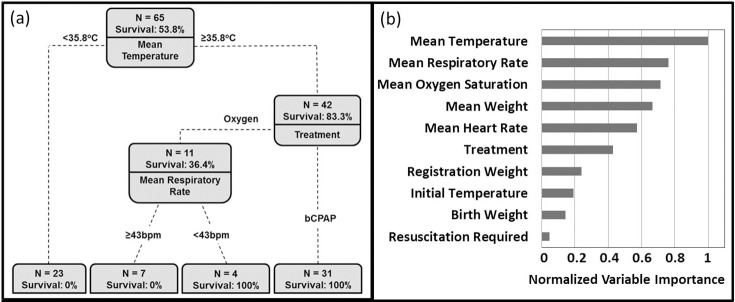
(A) Fitted decision tree predicting survival on the basis of clinical features for neonates diagnosed with RDS. The first node represents the mean temperature; no neonates with a mean temperature <35.8° C survived to discharge. (B) Ranked order of relative importance of the predictive variables for the root node. The top variable predicting survival is mean temperature over the duration of treatment.

Fig 1A shows the fitted classification tree for predicting survival to discharge for neonates with a primary diagnosis of RDS. Four groups of neonates were identified. None of the 23 neonates (0%) with a mean temperature during treatment less than 35.8° C survived to discharge regardless of whether they were treated with oxygen or bCPAP (group 1). All of the 31 neonates (100%) with a mean temperature during treatment of at least 35.8° C who were treated with bCPAP survived to discharge (group 2). Outcomes for neonates with a mean temperature greater than or equal to 35.8° C who were treated with nasal oxygen were predicted with one additional covariate, mean respiratory rate. None (0%) of the seven neonates meeting these criteria with a mean respiratory rate greater than or equal to 43 bpm survived to discharge (group 3), while all 4 (100%) of the neonates with a mean respiratory rate less than 43 bpm survived to discharged (group 4).

The top 10 covariates for splitting the root node of the fitted tree are ranked in order of importance in Fig 1B, with mean temperature ranking as the top covariate for predicting survival. The following two covariates, mean respiratory rate and mean oxygen saturation, are directly related to the treatment received as both should improve with appropriate treatment. Validation of the model via the bagging method with 100 bootstrapped trees revealed that although mean temperature was most often chosen as the first covariate to split the root of the tree (89%), it was not unique, with mean respiratory rate and mean O_2_ saturation being chosen as the first covariate for 9% and 2% of the trees, respectively. The model was further evaluated by calculating the probability of survival for each subject, using only the bootstrapped trees grown in the absence of that subject. This out-of-bag technique is not influenced by overfitting and indicates a high degree of prediction accuracy: The probability of correctly predicting survival was 97% (false-negative rate of 3%), while the probability of correctly predicting death was 90% (false-positive rate of 10%).

**Fig 2:**
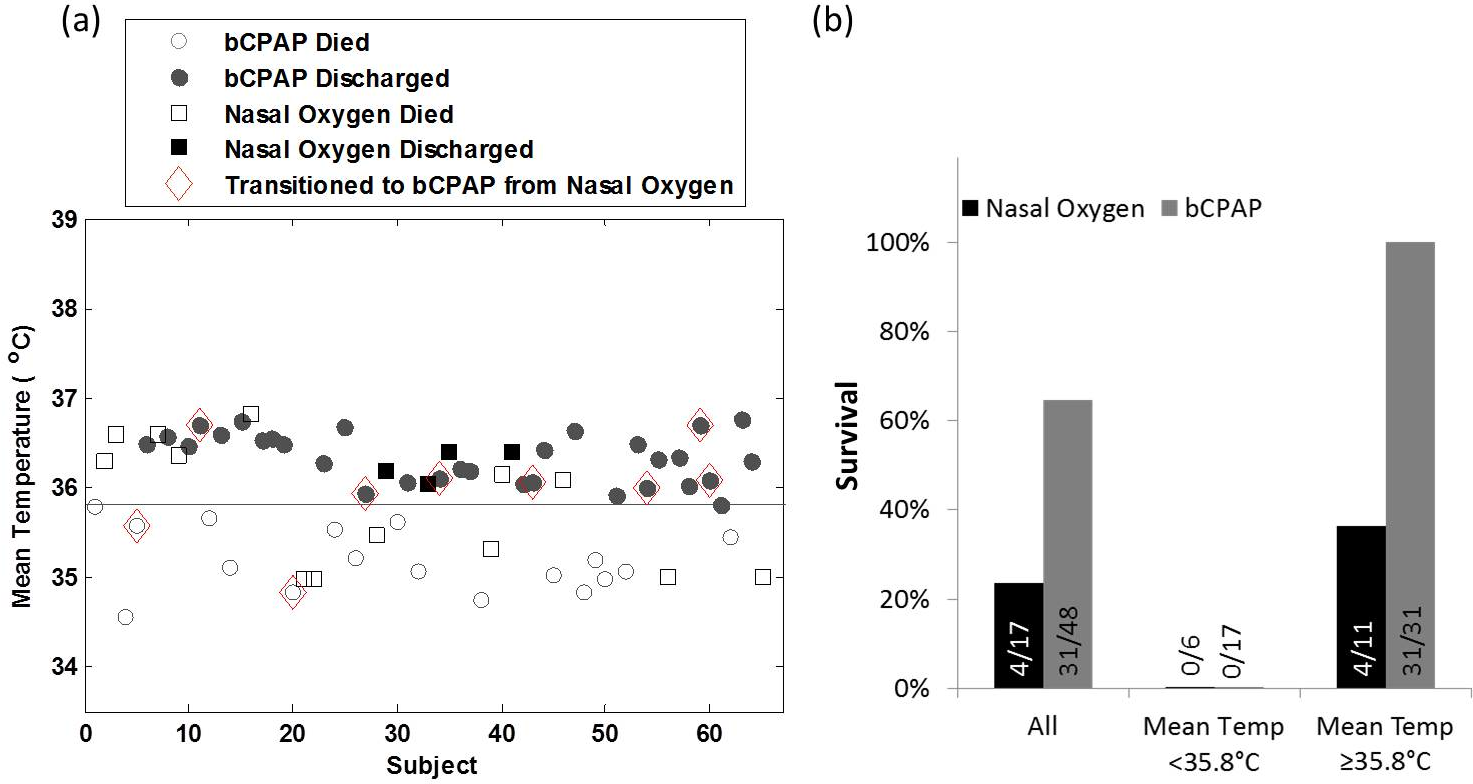
(A) The mean temperature for neonates diagnosed with RDS and treated with bCPAP (circles) or nasal oxygen (squares); filled symbols correspond to neonates who survived to discharge and open symbols correspond to neonates who died. Neonates initially treated with nasal oxygen, but later transitioned to bCPAP when a machine became available are designated with a red diamond. (B) Percent of neonates surviving stratified by treatment group for neonates diagnosed with RDS, and for neonates with a mean temperature above and below 35.8° C.

Fig 2 illustrates the relationship between mean temperature during treatment and the primary outcome of survival to discharge in greater detail. The mean temperature during treatment is shown in Fig 2A for each neonate with a primary diagnosis of RDS, coded by treatment group. None of the 23 neonates (0%) with a mean temperature of less than 35.8° C (as indicated by the horizontal line) survived to discharge. In contrast, 35 of the 42 neonates (83.3%) with a mean temperature greater than or equal to 35.8° C survived to discharge. Fig 2B compares rates of survival for neonates with RDS treated with nasal oxygen and bCPAP, stratified by mean temperature. Overall, 64.6% of neonates with RDS treated with bCPAP survived discharge, as compared to the 23.5% survival rate for those treated with nasal oxygen. However, the improvement in survival associated with bCPAP was greater for neonates with a mean temperature greater than or equal to 35.8° C; 100% of neonates treated with bCPAP survived to discharge compared to 36.4% for those treated with nasal oxygen.

**Fig 3:**
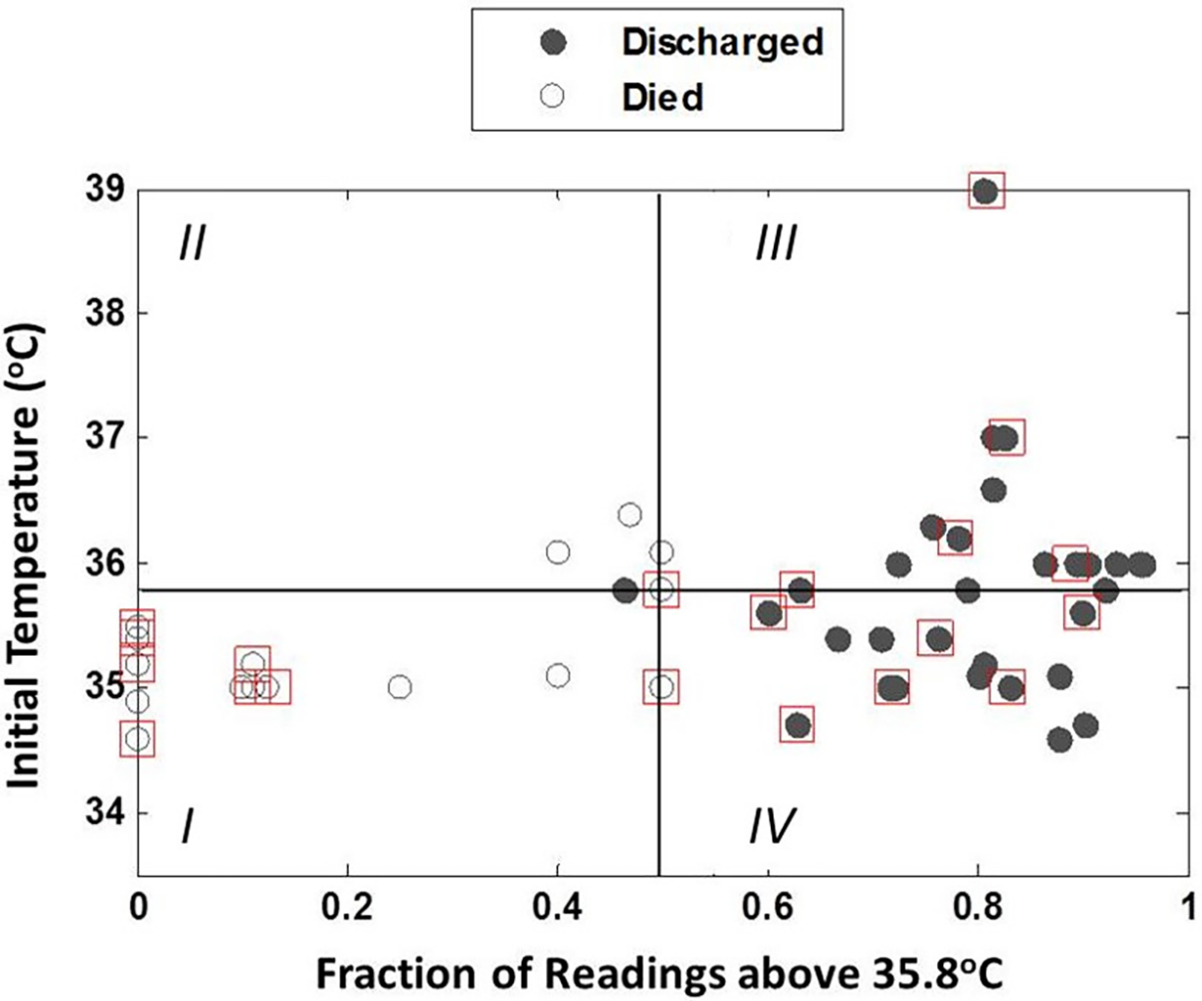
Initial temperature vs. the fraction of temperature measurements above 35.8° C for all neonates treated with bCPAP (N = 48). Filled symbols correspond to neonates who survived to discharge while open symbols indicate neonates who died. Neonates with a co-morbidity of sepsis are highlighted with a red square. Neonates who were admitted warm and became cold, or were admitted cold and remained cold (quadrants *I* and *II*) had poor survival rates, while neonates who were admitted warm and remained warm, or were admitted cold and became warm (quadrants *III* and *IV*) had high survival rates.

We examined the relationship between initial temperature and mean temperature during treatment on rates of survival to discharge for neonates diagnosed with RDS and treated with bCPAP. On average, the mean and initial temperatures for neonates who survived to discharge are significantly higher than those who did not (p = 0.025 and p<0.001, respectively, S1A Fig). However, the average standard deviation in temperature for neonates treated with bCPAP who survived to discharge was nearly identical to that experienced by neonates who did not survive (p = 0.98, $\bar{s}$ = 0.73 for both), with both groups experiencing relatively large temperature fluctuations throughout their treatment (S1B and S1C Fig). Fig 3 shows the initial temperature vs. the fraction of temperature readings above 35.8° C during treatment for each neonate diagnosed with RDS and treated with bCPAP. Symbols are coded to reflect the primary outcome as well as presence of co-morbidities, and a horizontal line is drawn at 35.8° C. Each quadrant of this graph represents a different clinical scenario. Subjects falling in quadrant I of the graph (lower left) are those who were initially and remained hypothermic; none of these 13 neonates (0%) survived to discharge. Similarly, subjects with an initial temperature above 35.8° C who became hypothermic had poor outcomes, with only one of these 5 neonates (20%) surviving (quadrant II, upper left). In contrast, all 30 neonates with more than 50% of temperature measurements above 35.8° C survived to discharge, regardless of whether their initial temperature was above or below this value (quadrants III and IV).

## Discussion

Hypothermia is a major challenge to the survival of newborns—especially for those that are sick or premature. This fact is not new; Morley reported cold injury to infants in the tropics nearly 60 years ago [27]. In 1981 Briend et al. wrote from Senegal that “deaths [from hypothermia] seem easy to avoid” [28], and yet neonatal hypothermia remains pervasive. It was estimated that in 2013, globally, hypothermia occurred in 32–85% of infants born in-hospital and 11–92% of babies born at home [2, 29]. In Uganda 79% of 300 newborns became hypothermic within 90 minutes of birth, of whom 64% had a birthweight >2500g [14]. In a Nepalese study 59% of term babies were hypothermic (<36.5°C) within 24 hours of birth [30]. In Zambia hypothermia was found in 73% of infants receiving standard care [31]. Though there is a seasonal element to hypothermia, neonates are vulnerable even in hot tropical environments [29].

As recently as 2014 it was estimated that proper thermal care management could avert 20% of neonatal deaths caused by preterm birth complications and 10% of deaths in full-term or moderately preterm babies caused by infection [19]. The WHO provides warnings with increasing severity as temperatures drop below 36.5°C [11]. Every 1°C drop in admission temperature below 36°C increases the risk of mortality by 28% and that of late-onset sepsis by 11% [7]. Hypothermia was associated with a two-fold increase in mortality for term babies, and hypothermic preterms had up to 30 times the mortality of normothermic infants [32]. In a Nigerian study, 37.6% of hypothermic infants died compared to 16.7% who were normothermic [15]. In a tertiary hospital in Eritrea, 9.4% of infants (100 of 1053) were admitted with a primary diagnosis of hypothermia, but hypothermia was a contributing factor in 22.9% of deaths [33].

The physiological effects of hypothermia in neonates are complex and ill understood; however, data from available studies suggest that hypothermia likely has an impact on respiratory distress. Hypothermia may lead to increased oxygen consumption and pulmonary vasoconstriction, increased respiratory distress and reduced surfactant release [34]. In premature infants, already low surfactant levels are further reduced by hypothermia and respiratory distress syndrome develops [35]. In a study from Taiwan, the mortality rate was higher in moderately hypothermic infants than in normothermic infants (18.5% vs 5.1%, p = .05) and more moderately hypothermic infants with RDS required surfactant (58.0% vs 39.2%, p = .006) [34].

Our study included preterm neonates with RDS who received bCPAP or nasal prong oxygen. Those who received bCPAP had improved survival. Survival was strongly influenced by hypothermia upon study registration and during treatment. If a neonate was initially hypothermic, or became and remained hypothermic, the outlook was bleak. All infants received standard thermal care. It may be that some infants were hypothermic because of the severity of their underlying illness, but the vicious cycle of hypothermia and RDS are difficult to unravel to be able to apportion blame. However, in many interventional studies to reduce hypothermia upon admission to the neonatal unit in higher resource settings, a corresponding improvement in respiratory outcomes has been noted [36–41].

Taken together, the results of our study and the pervasive challenge of hypothermia in low-resource settings suggest that successful implementation of CPAP to treat RDS in low-resource settings will require aggressive action to prevent persistent hypothermia. Babies can lose body heat at numerous points along the chain of care. The delivery room may be at less than the recommended 25–28°C [11]. Theatres are often air conditioned for the comfort of staff, but a change in the environmental temperature as small as 2°C can cause thermal stress in premature infants [42]. Simple interventions such as wrapping the dried baby in a warm cloth, as well as the use of a radiant heater while providing resuscitation, are very effective at preventing hypothermia [43]. If a radiant warmer is not available, Christidis et al. found that skin-to-skin care in the first hour after birth was as effective as a radiant warmer for term infants [44]; thus, the newborn should be transported to the nursery either skin-to-skin with mother or in a pre-warmed transporter.

Neonates in respiratory distress could be provided with heated, humidified air for respiratory support, but integrating heating coils within the breathing circuit is costly. Furthermore, humidification can be challenging in hot environments where access to clean water is unpredictable and fungal contamination is common. In this study, humidification was provided by using nasal saline drops, and the Pumani CPAP provided a mixture of oxygen from a concentrator and room air, which was continuously warmed by space heaters in the nursery. Studies in high-resource settings have shown improvements in admission temperature to the NICU when heated, humidified air is used to provide initial respiratory support for premature infants immediately after delivery [45, 46]. However, cutaneous heating methods, such as occlusive wraps, have in general been more effective in increasing the body temperature of premature infants in high resource settings [47, 48]. Further investigation is needed to determine the impact of heated, humidified air on the body temperature of neonates receiving CPAP treatment in low resource settings.

The main limitation of this analysis is the relatively small sample size. However, the original study did show significant improvement in survival for neonates diagnosed with RDS and treated with bCPAP as compared to those treated with nasal oxygen (p = 0.006) [24]. Moreover, prediction accuracy is the standard measure of performance for prediction models and validation of the fitted classification tree model presented here demonstrated a high degree of accuracy (> 90%) based on 65 patients. This classification tree analysis highlights the importance of temperature in predicting survival, showing zero survival for neonates with a mean temperature less than 35.8°C in this study. Furthermore, for those infants diagnosed with RDS and treated with bCPAP, the average temperatures of those who survived to discharge were significantly higher than the average temperatures of those who did not survive to discharge (p<0.001, S1A Fig). The charts collected for this study were on average over 99.5% complete, with no chart missing more than two readings for any of the vital signs collected during treatment. The danger of hypothermia is well known, and the importance of warmth is a well accepted principal. This study highlights the importance of addressing hypothermia in parallel with other interventions for newborn care. Nonetheless a larger study is needed to confirm these findings.

## Conclusion

A single intervention such as CPAP will not suffice in the absence of proper thermal care. Likewise, a patient with RDS who is kept warm but does not have access to breathing support also may not survive. An infant in respiratory distress requires comprehensive quality care including respiratory support and warmth in a clean environment.

## Supporting information

S1 Fig(A) Average initial and mean temperatures of infants treated with bCPAP who survived to discharge compared to those who did not, showing significantly lower initial (p = 0.025) and mean (p<0.001) temperatures for infants who did not survive. Temperature series versus bCPAP treatment time normalized with respect to the duration of treatment for infants (B) who survived to discharge and (C) for infants who did not survive showing similar temperature fluctuations with nearly identical average standard deviations for each group ($\bar{s}$ = 0.73°C) but lower overall temperatures for infants who did not survive.(TIF)
