# Supplementary figures and images for: Impact of hypothermia on implementation of CPAP for neonatal respiratory distress syndrome in a low-resource setting
Source: PLoS One. 2018 Mar 15;13(3):e0194144. doi: 10.1371/journal.pone.0194144 (PMC5854332; doi:10.1371/journal.pone.0194144)

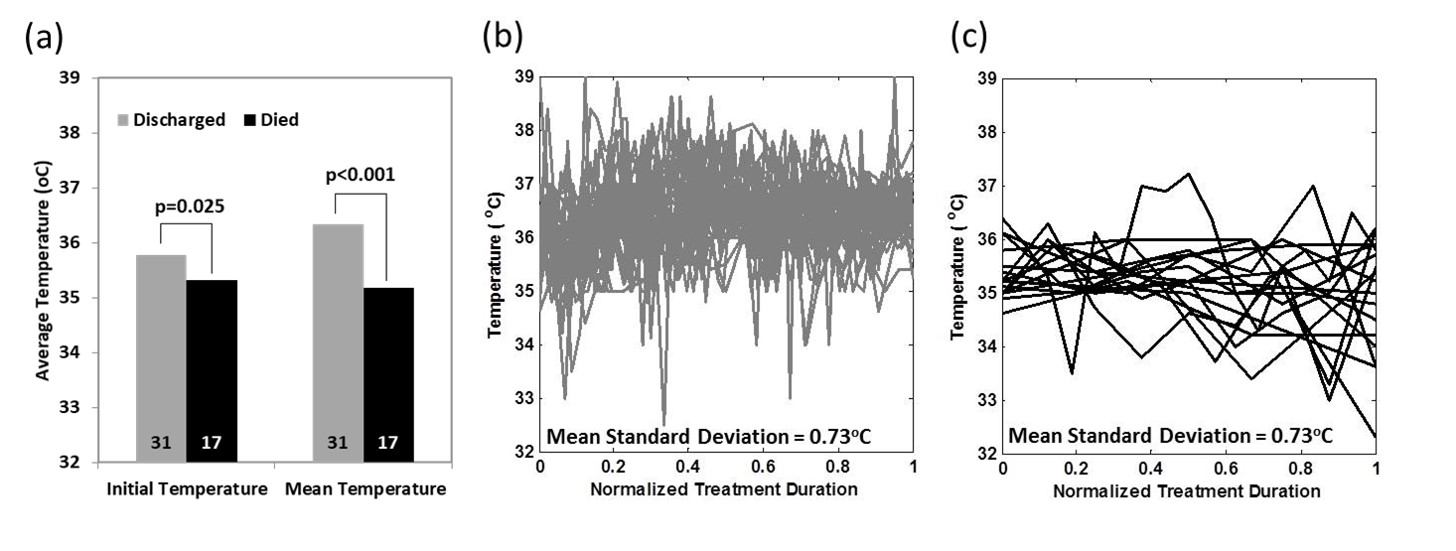

Supplement: S1 Fig — (A) Average initial and mean temperatures of infants treated with bCPAP who survived to discharge compared to those who did not, showing significantly lower initial (p = 0.025) and mean (p<0.001) temperatures for infants who did not survive. Temperature series versus bCPAP treatment time normalized with respect to the duration of treatment for infants (B) who survived to discharge and (C) for infants who did not survive showing similar temperature fluctuations with nearly identical average standard deviations for each group (s¯ = 0.73°C) but lower overall temperatures for infants who did not survive. (TIF) [file pone.0194144.s001.tif]
